# Bayesian Inference for Identifying Interaction Rules in Moving Animal Groups

**DOI:** 10.1371/journal.pone.0022827

**Published:** 2011-08-04

**Authors:** Richard P. Mann

**Affiliations:** Centre for Interdisciplinary Mathematics, Uppsala University, Uppsala, Sweden; Genentech Inc., United States of America

## Abstract

The emergence of similar collective patterns from different self-propelled particle models of animal groups points to a restricted set of “universal” classes for these patterns. While universality is interesting, it is often the fine details of animal interactions that are of biological importance. Universality thus presents a challenge to inferring such interactions from macroscopic group dynamics since these can be consistent with many underlying interaction models. We present a Bayesian framework for learning animal interaction rules from fine scale recordings of animal movements in swarms. We apply these techniques to the inverse problem of inferring interaction rules from simulation models, showing that parameters can often be inferred from a small number of observations. Our methodology allows us to quantify our confidence in parameter fitting. For example, we show that attraction and alignment terms can be reliably estimated when animals are milling in a torus shape, while interaction radius cannot be reliably measured in such a situation. We assess the importance of rate of data collection and show how to test different models, such as topological and metric neighbourhood models. Taken together our results both inform the design of experiments on animal interactions and suggest how these data should be best analysed.

## Introduction

Animal swarms produce complex patterns of behaviour that give the appearance of group intelligence, enabling animal groups to avoid danger, make collective decisions [1]–[4] and navigate more efficiently [2], [5], [6]. Self-Propelled Particle (SPP) models [7], [8], inspired by statistical physics, have demonstrated that these patterns can emerge through simple interactions between neighbouring particles.

The study of SPP models has shown that complex and realistic appearing patterns of collective motion can emerge from a wide variety of interaction rules, with different rules often producing similar patterns at the group level. Viscek *et al.* [9] classify three basic classes of group movement. In the *disordered* class the individuals move in a non-aligned fashion, moving randomly around a group centre. In the second class, *rotational* groups, individuals move around in a closed loop, orbiting a central point, with localised alignment but no global group movement. In *ordered* groups all individuals are aligned and the group moves coherently in a single direction. This apparent ‘universality’ in the group structures that emerge from different rules suggests that there are strong restrictions on the possible stable groups that can form from locally interacting moving individuals.

While classification of patterns can tell us a great deal about which group behaviour can emerge it complicates the identification of the specific interaction rules used by individuals. As noted by Li *et al.* [10], the emergence of a desired pattern in a simulation can not be taken as evidence of model correctness. Recent studies have shown that coherent group behaviour can emerge in bacteria [11] and even inorganic rods [12], [13] through physical contact alone. This suggests that animal interactions could be far simpler, or potentially far more complex than previously imagined. The emergence of similar group behaviour from differing rules means it is difficult to identify animal interaction rules by observing only the large scale group dynamics, since the measurable macroscopic properties of these groups such as group size and alignment may be matched using a variety of rules. As such, it is often only detailed analysis of the small scale motions of animals that can reveal the underlying interaction rules.

Identification of these rules, which may differ between individuals, between groups and between species, is the key question in many studies of behavioural ecology. Many of the emergent behaviours of groups can be understood without a detailed understanding of the underlying rules that generate them. However, questions regarding the evolution of social behaviour can be addressed by asking how interaction rules developed and whether the same rules evolved across different species. This necessarily requires methods to infer these rules, which analysis of the large scale behaviours of different models often can not provide.

Recent work has addressed not only which rules are necessary, and at what strengths, to produce realistic behaviour, but also what determines who interacts with whom. The debate has focused on how the neighbourhood of each individual, the other animals it interacts with, should be defined. Traditional SPP models allowed each individual to interact with others within some fixed *geometrical* distance [7], [8]. More recent work has considered a *topological* definition [14], [15], allowing each individual to interact with a fixed number of closest neighbours, independent of the absolute value of the geometrical distance between them. Other research has looked at directed and hierarchical models of leadership and following in groups [2], [4], [16]. A key problem then in examining empirical data is determining the degree to which it supports different interaction models.

As the technology for tracking animals in motion has improved, through the use of video analysis [17], [18], Radio Frequency Identification (RFID) [19]–[21] and Global Positioning Satellite (GPS) tracking [22]–[25], the possibility has emerged of identifying interaction rules by observing the movements of individuals within the collective. Recent studies have shown that the parameters of animal swarm models can be matched to recorded data, either by regression analysis to minimise the difference between observed and predicted movements [26] or by more empirical analysis of spatial correlations of position and direction within the group [27]. Where there is an explicit ‘loss-criterion’, such as the predictive error, evaluating this criterion over a range of parameter values can identify the best-fit parameter set. Likewise hypothesis testing can be performed by determining which mimises this criterion.

An important aspect of model fitting is knowing the uncertainty associated with inferred parameters. This is especially important when considering data collected from animal groups. Experimental limitations mean data often consist of a small number of observations, and high levels of biological variation mean that these data are often noisy. Furthermore these observations are typically taken from stable group structures where the configuration of the neighbouring animals changes only slowly over time. For example, fish often form stable rotating mills with a relatively constant radius, making the interactions between individuals over a wider range of distances impossible to examine. Under these circumstances it is important to identify and acknowledge the uncertainty in estimates of, for example, the interaction radius of the fish. Animal interaction data is often collected as part of an iterative process whereby new data becomes available with each new experiment. If we are to make meaningful comparisons between this series of experimental outcomes we must be able to say how certain we were about our conclusions at each stage.

Model and parameter uncertainty are best understood through a fully probabilistic method. Bayesian inference uses the probability of the data, the likelihood, to provide a complete probability distribution over the model parameters. This probability distribution can be iteratively updated as more data becomes available, providing an easily interpretable measure of model fit and uncertainty that can be consistently updated in the light of new experimental evidence.

In this study we pursue two goals. Firstly to demonstrate a fully Bayesian methodology for parameter estimation and hypothesis testing in SPP models. Secondly to investigate how inference is affected by the possible restraints technology places on data collection. We will determine the effect of having very few recorded data points, of including significant observation noise in the data and of having temporal resolution which differs from the characteristic timescale of the observed system. We will also show how any issues these restrictions cause can be ameliorated through adaption of the inference procedure.

## Results

**Figure 1:**
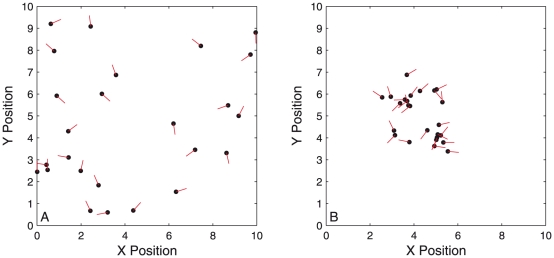
Example of the simulated system, with random initial configuration A and the steady state rotating mill solution B. Motion is in the direction of the red lines from each particle. Within the rotating mill there are typically particles moving both clockwise and anti-clockwise simultaneously.

We generated simulated data from the two-dimensional SPP model described below in ‘Methods and Materials’, with 25 particles and with parameters chosen to allow the particles to converge to a steady state solution of a rotating mill, a solution common to many models of collective motion [8], [9], [28], [29]. Figure 1 shows an example of the system, with the particles in their initial random configuration (Figure 1 A) and the steady state rotating mill solution (Figure 1 B). An observation from our simulations which may be unrealistic for natural groups is the co-existence of clockwise and anti-clockwise moving particles within the rotating mill. This has previously been observed in a similar model by Strömbom [29] and is a consistent feature across our simulations within the range of parameters and model variations investigated in this work.

### Inference in random and steady-state configurations

We begin by establishing the ability of the inference procedure to identify the correct values of parameter values in a known model. We do this by observing convergence of the parameter probability distribution to the known values used to simulate swarm data. We do this under two ‘experimental regimes’ – with particles in an initially random configuration (Figure 1 A) and with particles moving in a steady-state configuration of a rotating mill (Figure 1 B). Using the data analysis methodology outlined in ‘Methods and Materials’ we perform inference using the first recorded time-step of the data, then the second time-step, continuing up to the tenth time-step of the simulation, using equation (9) to iteratively update the probability distribution over the parameters. We record the mean and standard deviation of the probability distribution over each parameter to observe convergence to the true value. We also record the information entropy of the full joint distribution of all parameters to give a single value to express the remaining uncertainty in the model parameters. We repeat the same analysis for the final ten recorded time-steps, after the system has converged to the steady-state solution (the rotating mill).

**Figure 2:**
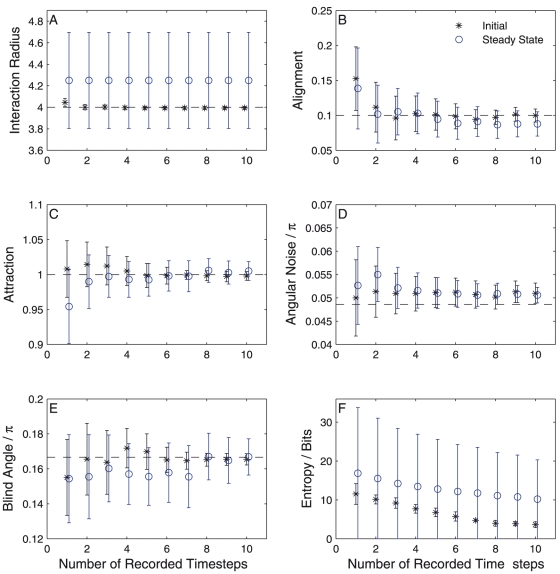
Convergence of the parameter probability distribution toward the true values over ten recorded time-steps. Points represent the mean of the probability distribution while error bars represent the standard deviation. Black stars show results using data recorded from a random initial particle configuration. Blue circles show results using data taken from the steady-state rotating mill configuration. All results are averaged over five independent trials.

Figure 2 A–F shows how the mean and standard deviation of the probability distribution for each parameter and the entropy changes with increasing data size for both the random and steady-state configurations. As more time-steps are included in the analysis all parameters converge rapidly towards their indicated true values when using data taken from the start of the simulation (black stars). Using data taken from the steady-state (blue circles) markedly reduces the rate of convergence for all parameters. This is consistent with the idea that the data now contain fewer different configurations of the particles and are thus less informative. The extreme example of this can be seen in the distribution of the interaction radius (Figure 2 A). The black stars show the interaction radius is well identified after just a couple of time-steps from the random configuration. Conversely, the blue circles show that using steady-state data the distribution of this parameter is practically unchanged from the prior probability distribution and shows no convergence towards the true value. This is explainable as the result of all particles within the rotating mill being within a single interaction radius. Whilst this does not mean that all particles interact (due to the blind angle) it means the data analysis is unable to ‘see’ the effect of particles being further separated and therefore can not judge whether interactions cease beyond some range. All that can be said is that the interaction radius is *above* some value that would lead to particles within the mill being disconnected.

The entropy of the joint parameter probability distribution (Figure 2 F), calculated via equation (10), describes the total uncertainty in the parameter estimate. This shows a roughly linear decrease for both initial- and steady-states. This indicates that within this small time region each new time-step adds approximately the same levels of information to the inference. As expected from the individual distributions we see that the entropy in the steady-state is consistently higher than the initial-state and decreases more slowly, indicating slower convergence of the inference.

### The effect of noise

The noise parameter in the model represents unexplained variation – variation that the model cannot account for. In real systems this variation comes from two sources. One is observation noise, due to the limits of the technology to accurately track real animals. The other is the deviation of the animal's true behaviour from the model predictions. In our simulation data these two effects are approximated by the addition of random angular noise to the heading of each particle at each time-step.

**Figure 3:**
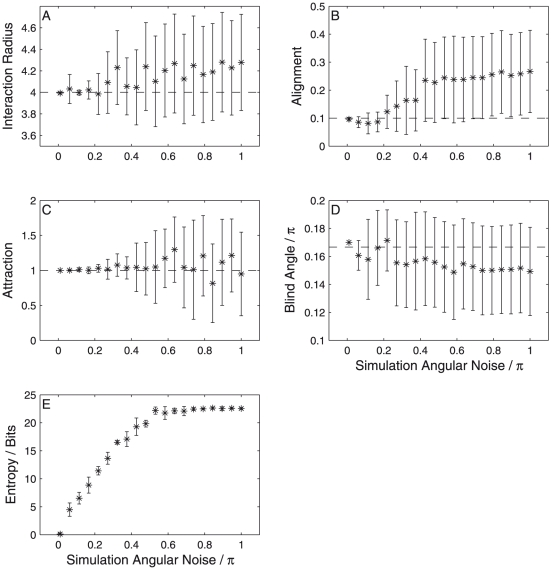
Divergence of the parameter probability distribution away from the true values with increasing simulation noise. Points represent the mean of the probability distribution while error bars represent the standard deviation. Results are from data taken from a random initial configuration and are averaged over five independent trials.

Excessive noise from either of these two effects may make inference difficult in real systems. To test the effect of increasing noise we performed inference on simulated data with varying noise levels. We used ten recorded time-steps from each simulation to infer the probability distribution over the parameters and tracked the mean and standard deviation of each parameter along with the entropy of the distribution. Figure 3 shows that every model parameter become more uncertain and more divergent from the indicated true value as the noise level is increased. The quality of inference is rather sensitive to the noise level, with some parameters, notably the blind angle (Figure 3 D), rapidly approaching the *a priori* distribution as the noise is increased. Other parameters undergo a non-linear increase in uncertainty at some critical value of the noise. For example, the attraction parameter is inferred well for noise levels below approximately 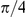 but then rapidly deteriorates (Figure 3 C).

The clearest indication of how inference quality is determined by the noise is given by the entropy of the parameter distribution (Figure 3 E). The entropy of the distribution first increases linearly with noise, showing that information is being rapidly lost. With noise levels above approximately 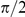 the entropy plateaus at a value equal to the entropy of the *a priori* distribution – the first guess at the parameters before any data are seen. Therefore we can establish that in this system no useful inference is possible when the angular noise – the variation in movement that can not be modeled – exceeds 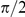. This is consistent with intuition since this degree of noise allows for a full range of angular direction changes within a semi-circle ahead of the particle through random chance alone.

### Effect of data collection rate

In real systems data collection rates are determined by the selected tracking technology. Modern tracking solutions offer very high frequency data collection, often many data points per second, that may greatly exceed the characteristic timescale over which animals react to each other and change direction. Here we show that it is important to consider the temporal resolution of the data relative to the characteristic timescale by performing inference on simulated data sets with differing data collection rates.

**Figure 4:**
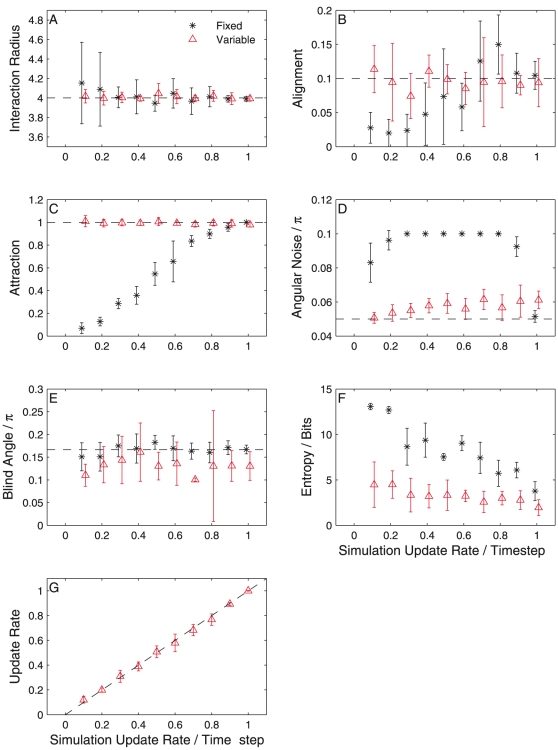
Variation of the parameter probability distribution with varying update rate in simulation, in the case where inference assumes an fixed update rate of unity (black stars) and the case where the update rate is variable and inferred (red triangles). Points represent the mean of the probability distribution while error bars represent the standard deviation. Results are from data taken from a random initial configuration and are averaged over five independent trials.

We simulate a data collection rate above the characteristic timescale by providing for each particle in the simulation to update at each time-step with some fixed probability, 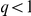, such that only a subset of the particles update their direction on each time-step. Those particles that do not update retain their previous direction, but still with the addition of random noise. We then investigate the consequences of not accounting for the rapid sampling rate by inferring the parameters of the system assuming an update probability of unity. We observe how the probability distribution of each parameter and the entropy change as the update rate is varied between zero and one. In Figure 4 A–E we plot the variation in the mean and standard deviation of the probability distributions with 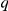 based on ten observed time-steps of simulated data in two cases. The first is when we make the assumption during inference that 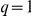 (black stars). This shows the consequences of failing to consider the possibility of a lower update rate. The second case is when we allow the update rate to be inferred simultaneously with the other parameters (red triangles). For the case where the update rate is assumed to be unity we see that every distribution converges towards the indicated true value as 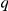 is increased towards one. The distributions of different parameters show differing behaviour as 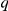 is decreased. The parameters associated with the structure of the neighbourhood – the interaction radius (Figure 4 A) and the blind angle (Figure 4 E), show little consistent bias and simply become rather more uncertain with lower 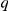. This is consistent with there being fewer updates for the model to learn the neighbourhood from, which effectively represents less available data. On the other hand, the alignment parameter (Figure 4 B) and attraction parameter (Figure 4 C) show consistent and monotonically increasing biases as 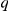 is varied. In the case of the attraction parameter this bias is very well defined and almost linear, while the alignment parameter is less consistent, likely due to its proximity to zero. What this shows is that with fewer updates each particle changes its direction less *per time-step* than would otherwise be the case. If the inference procedure believes the update rate to be one then it must compensate by making the respective forces weaker. These adjustments necessarily make the model less accurate at predicting the fine-scale motions, since it either updates too strongly when no update should occur or too weakly when one should. To account for this we infer a great deal more noise (Figure 4 D) when 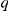 is less than one.

This bias, imposed by collecting data at a rate faster than the characteristic timescale, is compensated by inferring the update rate simultaneously. Adding this parameter to the model and performing the inference again we find that we consistently infer the correct value of 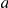 and 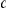 (Figure 4 B and C, red triangles). The distribution of the interaction radius (Figure 4 A) and the blind angle (Figure 4 E) remain unbiased and broadly unchanged, with rather less uncertainty in the interaction radius and somewhat more on the blind angle. The update rate itself is inferred with very high accuracy (Figure 4 F). Since the model now fits the data better we infer a lower amount of noise, much closer to the true value (Figure 4 D). The entropy of the parameter distribution (Figure 4 G) is consistently lower when 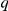 is inferred. The entropy in both cases converge as 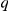 approaches unity.

### Model selection

**Figure 5:**
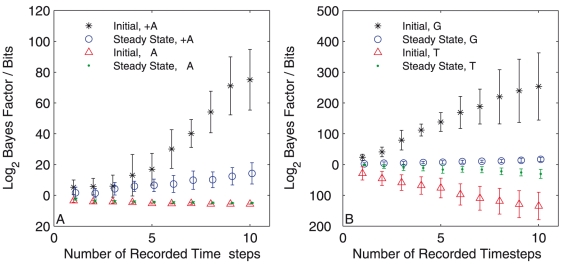
Two model selection scenarios. Panel A shows the log Bayes factor for a model including an alignment term versus a model without such a term. Each curve shows the variation in the Bayes factor as the quantity of data is increased and represents a different simulated data set. Positive values indicate support for an alignment model, while negative values indicate support for a no-alignment model. Black stars and blue circles show results derived from simulations including an alignment term (+A) and represent the initial and steady-state configurations respectively. Red triangles and green dots show the same calculations based on simulations where an alignment force was not included (−A). Panel B shows similar results from a model selection between a geometrical and a topological model. Positive values indicate support for an geometrical model, while negative values indicate support for a topological model. Black stars and blue circles show the Bayes factor calculated based on simulations where a geometrical scheme was used (G) and represent the initial and steady-state configurations respectively. Red triangles and green dots show the same calculations based on simulations where a topological scheme was used (T). In all four cases the correct model is increasingly supported as more data are analysed. All results were averaged over five independent trials.

We examined two model selection scenarios. Firstly, since one of the primary advantages of Bayesian inference is the automatic selection of an appropriate model complexity (number of free parameters) and the avoidance of overfitting (see Methods and Materials: Data analysis), we examine whether our method can identify when a potential force factor is absent. In this case we simulate data using two subtly different SPP models, one including an alignment force and one where alignment is absent. Both models converge to the same large scale behaviour, the rotating mill formation, and therefore cannot be trivially distinguished by observations of the macroscopic motion. We calculate the Bayes factor to determine how well each data set supports a model including an alignment term as opposed to a no-alignment model, using equation (11) and defining the alignment model as model 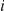 in the numerator and the no-alignment model as model 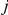 in the denominator. We perform this calculation with data recorded in both the random initial configuration and the steady-state to determine if the configuration of the swarm has an influence on the power of the selection procedure. Figure 5 A shows that when an alignment model is used to generate the data we see rapidly increasing support in the Bayes factor for an alignment model in both the initial (black stars) and steady-state (blue circles) configurations. When data are simulated from a non-alignment model we also see decreasing support for the inclusion of an alignment force in both the initial state (red triangles) and the steady-state (green points), though here the absolute value of the Bayes factor is much lower. This is likely to be because a no-alignment model can be accurately approximated by a model allowing alignment simply by reducing the alignment parameter to zero, so only a complexity penalty is left to discern between the models.

Secondly, in line with current interest in the literature [14], [15] we aimed to determine whether a given data set was simulated from a topological or a geometrical model. We simulate data from two similar SPP models, this time differing in the definition of each particles neighbourhood. In one model neighbours are selected according to geometrical scheme and in the second a topological scheme is used. As before we check the the rotating mill formation is the steady-state for each model and evaluate the Bayes factor in both the initial and steady-state configurations, using equation (11) and defining the geometrical model as model 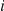 in the numerator and the topological model as model 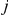 in the denominator. Again we see strong support for the correct model in each case in Figure 5 B. In line with our earlier findings, for both the complexity selection and the neighbourhood scheme selection we see clearer support for the correct model when using data from an initially random configuration. In the neighbourhood scheme selection we see a striking difference between this and the steady-state data, which is due to a lack of variation in neighbour positions whilst in the steady state. This is further confirmation that inference is best served by collecting data from a wide variety of swarm configurations.

## Discussion

We have demonstrated a fully Bayesian approach to parameter inference and model selection in models of collective animal motion. The probability distribution over models and parameters allows us to determine the conditions under which inference is successful and the degree of accuracy in fitting. Parameters relating to attraction and alignment can be measured with a high degree of accuracy from a comparatively small quantity of data. Eriksson *et al.* [26] used 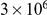 observations to estimate the parameters in their simulations. By comparison, here we could reliably estimate the parameters from a total of 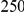 individual observations. It is likely that Eriksson *et al.* could have made reliable estimates from fewer data points, and one should be careful in comparing analyses on different models, but this shows that a realistic number of experimentally collected data points is sufficient to make accurate parameter inference. The specific number of observations required is less important than the ability to estimate the parameter uncertainty, which means we can determine when sufficient data have been collected. Our method allows for more direct calculation of parameter uncertainty and iterative inclusion of additional data than Eriksson *et al.* [26]. This is particularly relevant in cases such as the three-dimensional starling flock data set collected by the STARFLAG project [14], [30], [31] where large numbers of individuals are tracked for only a few frames. Given the correct model for starling behaviour we would be able to accurately estimate the interaction parameters from these limited observations. The trade-off of this added power in the analysis is a greater computational load. In principle our methods could be applied to large scale groups with thousands or more members over long time periods. However, the high processing demands of the full Bayesian analysis has limited us to a small group in this instance. Bayesian methods become extremely intensive for models with many free parameters. As the quantity of data becomes very large we would expect our methods and those of Eriksson *et al.* [26] to converge as the distribution over the parameters becomes more peaked around the true value. Where the parameter uncertainty is low the added computational speed of other methods may in some cases be preferred.

The probability distribution over parameters also allows us to identify when our parameter estimation is poor. In particular systems that are too stable present a limited number of particle configurations, which then lead to large uncertainties in model parameters. For example we found that stable milling configurations, which are a common feature of experimentally observed of groups [8], prevented inference of the interaction radius. Since in the random configuration we were able to estimate the interaction radius and improve our estimates of other parameters our results suggest that some degree of disorder can be useful for determining behavioural rules since it provides the animals with a wider range of situations to respond to. We note that it can be difficult in practice to manufacture random initial starting conditions without disturbing the animals' natural behaviour. However, the iterative nature of Bayesian updating (see equation (9)) allows a sequence of data collected from different natural configurations to be incorporated one-by-one to improve model estimates. For example, fish placed in a sequence of tanks of varying shape may exhibit different stable modes of collective movement, which can then aid the estimation of interaction rules when these data are combined.

Large quantities of random variation, either through observation noise or through overly simplified modeling eventually prevent useful inference. We have shown that the effect of noise in the system is generally non-linear. We found that while the loss of information, as measured by the information entropy of the full parameter distribution, was linearly dependent on the quantity of noise, each parameter individually had a non-linear increase in uncertainty beyond some value of the simulation noise. This demonstrates the importance of finding the right balance between the time and expense of data collection and the quality of the data. Since the quantity of data was found to have a more linear effect on inference uncertainty our results suggest it may often be better to collect fewer, higher quality data than to record movements inaccurately over longer times.

Our results demonstrate that a mismatch between the data collection rate and the characteristic time scale over which animals change direction leads to biased estimates of parameter values. However, in our model the inferred values of parameters associated with the interaction structure (who interacts with whom) were not biased when data were collected faster than the characteristic time scale. Parameters associated with the strength of forces such as attraction and alignment were linearly biased as a function of the data collection rate. Since the effect of these forces is multiplied by the number of time steps within any unit of time this suggests that the behaviour of the particles is still consistent with this inference. We also find that explicitly including a variable rate of direction change in the inference procedure can remove this bias and reduce the inferred level of noise, making inference of each parameter more robust. This comes at the cost of further extending the computational cost for inference and is most applicable when the direction changing rate is much lower than the data collection rate.

Of potentially even greater importance than parameter estimation within models is determining which of a variety of models best captures the animals' behaviour. Recent data collected from starling flocks has challenged the standard geometric model of interactions, suggesting that a topological model better accounts for the global structure of flocks. These results are not, however, based on looking at the interactions between the birds. Using the methods proposed here, even with limited available data on individual interactions, we are able to determine when the data favours one model over the other. This is just one example of distinguishing between models which in terms of their global pattern belong to the same universality class [9]. For example, Strömbom [29] uses a similar model to ours, but without an alignment term to produce a rotating mill. The Bayesian model automatically eliminates unnecessary parameters and thus when we analyse data simulated from this alignment-free model we can infer the simpler attraction-only model.

The study of collective motion is entering an exciting phase where global observation of animal groups is being complemented by fine-scale individual tracking. The physicists' ideal of universality in group behaviour must be reconciled with the biologists' aim of identifying the differences between animal species. By allowing inference of different particular interaction structure within the same global group behaviour our method presents a way out of this dichotomy.

## Materials and Methods

The ‘data’ we analyse comes from simulations of swarms using an SPP model adapted from Strömbom [29]. Particles experience inertia, align with their neighbours' direction of motion and are attracted to the centre-of-mass of the neighbouring particles. The neighbourhood is defined to include all particles within some perceptual range, but limited by a blind angle that prevents each particle from ‘seeing’ proximate particles within a region behind it. The neighbourhood can also be defined through a topological distance, which we explore later. At each timestep every particle updates its current direction and position according to these rules, the relative strengths of which are determined by a set of adjustable parameters.

### Equations of motion

We use the current positions 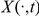 and headings, 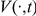 of the particles to determine which particles are in the neighbourhood, 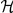, of particle 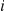. From this neighbourhood we calculate the alignment vector, 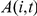 and centre of mass vector 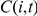, which we normalise to unit length. In the default geometrical model ‘neighbours’ are those particles within some euclidean distance 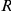. In the topological model the ‘neighbours’ are the closest 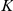 particles not excluded by the blind angle. Particles move in an 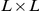 sized space, and move over periodic boundary conditions.
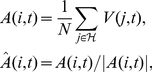
(1)

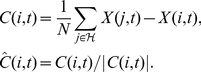
(2)We update the direction based on these forces, modulated by parameters 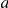 and 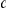 respectively. The direction vector will subsequently be normalised to unit length, therefore we can set the inertial parameter to one without loss of generality.
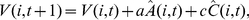
(3)Random noise, 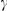 is added to the new direction vector, to represent the effect of observation inaccuracy and unexplained variation in the movement. We use angular noise drawn from a wrapped Gaussian distribution.
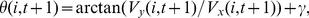
(4)

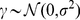
(5)

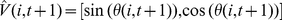
(6)Finally the position of particle 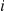, is updated according to its new direction. The speed of all particles is constant and identical, 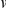.
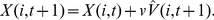
(7)Variable speed could be introduced as an additional element in the model. Our experience suggests that variation in speed that is not directly correlated with direction changes does not change the results of our analysis.

### Data analysis

Inference of model parameters involves determining the probability distribution of the parameter values based on observed data. The first step is to define the *likelihood* function – the probability of a set of observations conditioned on a known model, 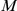 and parameter set 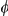. Since the positions of the particles are completely determined by their headings (speed being constant), we need only examine the probability of the changes in direction. The system is Markovian and the random noise is added to each particle independently. Therefore the likelihood is a product of terms, multiplying over particles and timesteps. Each term is a Gaussian probability determined by the difference between the new heading, 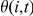 and the expected heading, 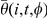 calculated from the known parameters, 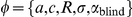. If 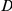 is the data, representing all the recorded direction changes of every particle within an experiment then,
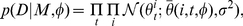
(8)where the notation 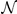 is used to denote the wrapped Gaussian probability density function, with variance 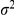. Since the system is Markovian the probability of the observations depends only on the value of the model parameters, 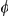, and not directly on any previously observed data.

Bayesian inference (see [32]) is based on iteratively updating the probability distribution of the parameters, 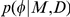 based on the likelihood of the new observations, evaluated using Bayes' rule. Let the current data, 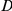, be composed of previously recorded data, 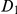, and new data 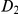, then,
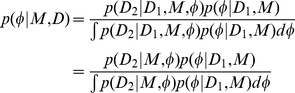
(9)Where 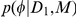 represents the probability distribution over the model parameters *before* observing the new data. Before any data is observed this is typically set to be a uniform distribution over a broad range of sensible parameter values. Bayes' rule therefore allows us to incorporate each new piece of evidence as it appears to refine our estimates of 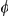. We have used the Markov property to remove the dependence of 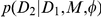 on the previous data, since the probability of observations are only connected by the model parameters.

The probability distribution of a single parameter can be concisely summarised by the mean and standard deviation in cases where the distribution is approximately symmetrical around the mean. The uncertainty of the distribution, for either a single parameter or for the joint distribution of many parameters, can also be quantified by the Shannon information entropy, 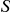 [33]. Entropy is a functional of the probability distribution. For a finite set of sample parameter values, 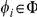, 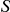 is calculated as,
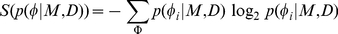
(10)The entropy of a parameter distribution represents the expected information gained by learning the true value of the parameters, or equivalently the expected information lacking due to not knowing these true values. The change in 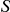 each time new data is added to the inference therefore measures how informative the new data is. As the distribution converges to a single point estimate the entropy tends to zero, expressing that no new information can be acquired.

Alternative models are readily compared by evaluation of the Bayes factor, 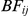 – the relative probability of the all observed data conditioned on the two different models, 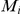 and 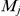. Model parameters are marginalised by integration so that the models themselves are directly compared. We can also calculate the Bayes factor iteratively, utilising the Markov property to remove direct dependences between data sets.
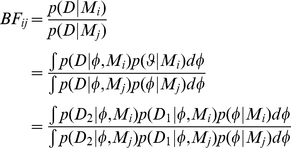
(11)This allows us to compare *classes* of models, as opposed to determining the correct model parameters. Hence, for example, we can infer if the data support a geometrical or a topological model, or whether certain model aspects such as alignment, attraction or the blind angle should be included at all. The integration over model parameters provides a quantitative incorporation of the principle of Occam's razor, automatically penalising overly-complex models by decreasing the prior probability mass for any particular set of parameter values since the prior must sum to unity over the complete space (see Mackay [34] for more details). The Bayes factor gives the relative probability of models 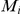 and 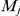 if both models are equally probable *a priori*. Therefore we can interpret the Bayes factor as the extent to which the data support one model over the other.

Matlab source code implementing the methods described is provided alongside this paper.
